# Translational and clinical comparison of whole genome and transcriptome to panel sequencing in precision oncology

**DOI:** 10.1038/s41698-024-00788-3

**Published:** 2025-01-10

**Authors:** Irina A. Kerle, Thomas Gross, Anja Kögler, Jonas S. Arnold, Maximilian Werner, Jan-Niklas Eckardt, Elena E. Möhrmann, Marie Arlt, Barbara Hutter, Jennifer Hüllein, Daniela Richter, Martin M. K. Schneider, Mario Hlevnjak, Lino Möhrmann, Dorothea Hanf, Christoph E. Heilig, Simon Kreutzfeldt, Maria-Veronica Teleanu, Evelin Schröck, Daniel Hübschmann, Peter Horak, Christoph Heining, Stefan Fröhling, Hanno Glimm

**Affiliations:** 1 Department for Translational Medical Oncology, National Center for Tumor Diseases Dresden (NCT/UCC), a partnership between DKFZ, Faculty of Medicine and University Hospital Carl Gustav Carus, TUD Dresden University of Technology, and Helmholtz-Zentrum Dresden-Rossendorf (HZDR), Dresden, Germany; 2 Translational Medical Oncology, Faculty of Medicine and University Hospital Carl Gustav Carus, TUD Dresden University of Technology, Dresden, Germany; 3 German Cancer Consortium (DKTK), partner site Dresden, Dresden, Germany; 4 Core Unit for Molecular Tumor Diagnostics (CMTD), National Center for Tumor Diseases (NCT), NCT/UCC Dresden, a partnership between German Cancer Research Center (DKFZ), Faculty of Medicine and University Hospital Carl Gustav Carus, TUD Dresden University of Technology and Helmholtz-Zentrum Dresden-Rossendorf (HZDR), Dresden, Germany; 5 Institute for Clinical Genetics, University Hospital Carl Gustav Carus at TUD Dresden University of Technology and Faculty of Medicine of TUD Dresden University of Technology, Dresden, Germany; ERN GENTURIS, Hereditary Cancer Syndrome Center Dresden, Germany; National Center for Tumor Diseases Dresden (NCT), NCT/UCC Dresden, a partnership between German Cancer Research Center (DKFZ), Faculty of Medicine and University Hospital Carl Gustav Carus, TUD Dresden University of Technology and Helmholtz-Zentrum Dresden - Rossendorf (HZDR), Germany; German Cancer Consortium (DKTK), Dresden, Germany; German Cancer Research Center (DKFZ), Heidelberg, Germany, Max Planck Institute of Molecular Cell Biology and Genetics, Dresden, Germany; 6 Department of Internal Medicine I, University Hospital Carl Gustav Carus, Dresden, Germany; 7 Else Kröner Fresenius Center for Digital Health, Technical University Dresden, Dresden, Germany; 8 Computational Oncology Group, Molecular Precision Oncology Program, NCT Heidelberg and DKFZ, Heidelberg, Germany; 9 German Cancer Research Center (DKFZ) Heidelberg, Translational Functional Cancer Genomics, Heidelberg, Germany; 10 Division of Translational Medical Oncology, National Center for Tumor Diseases (NCT) Heidelberg and German Cancer Research Center (DKFZ), Heidelberg, Germany; 11 Department of Hematology, Oncology and Rheumatology, Heidelberg University Hospital, Heidelberg, Germany; 12 German Cancer Consortium (DKTK), Heidelberg, Germany; 13 Innovation and Service Unit for Bioinformatics and Precision Medicine (BPM), DKFZ, Heidelberg, Germany; 14 Pattern Recognition and Digital Medicine Group (PRDM), Heidelberg Institute for Stem Cell Technology and Experimental Medicine (HI-STEM), Heidelberg, Germany; 15 Institute of Human Genetics, Heidelberg University, Heidelberg, Germany

**Keywords:** Molecular medicine, Translational research, Cancer genomics

## Abstract

Precision oncology offers new cancer treatment options, yet sequencing methods vary in type and scope. In this study, we compared whole-exome/whole-genome (WES/WGS) and transcriptome sequencing (TS) with broad panel sequencing by resequencing the same tumor DNA and RNA as well as normal tissue DNA for germline assessment, from 20 patients with rare or advanced tumors, who were originally sequenced by WES/WGS ± TS within the DKFZ/NCT/DKTK MASTER program from 2015 to 2020. Molecular analyses resulted in a median number of 2.5 (gene panel) to 3.5 (WES/WGS ± TS) treatment recommendations per patient. Our results showed that approximately half of the therapy recommendations (TRs) of both sequencing programs were identical, while approximately one-third of the TRs in WES/WGS ± TS relied on biomarkers not covered by the panel. Eight of 10 molecularly informed therapy implementations were supported by the panel, the remaining two were based on biomarkers absent from the panel, highlighting the potential additional clinical benefit of WGS and TS.

## Introduction

Over the last 15 years, the effectiveness of targeted drugs for different cancer entities that show either the same genetic alterations or surface-bound receptors has increasingly led to cross-entity systemic therapy approaches, making precision oncology a fast-growing field^1,2^. The poly ADP ribose polymerase (PARP) inhibitor olaparib has been approved for the treatment of four different tumor types in the presence of BRCA1 or BRCA2 mutations^3–6^, the PD-1 inhibitor pembrolizumab has been approved for the treatment of solid tumors with microsatellite instabilities or mismatch repair deficiencies^7^ and high TMB^8^, and the antibody-drug conjugate trastuzumab deruxtecan has shown clinical efficacy in a variety of HER2-positive solid tumors^9–11^. Although molecular diagnostics are already the standard clinical practice for cancers with recurrent driver alterations, such as cholangiocarcinoma, melanoma, non-small cell lung, breast, ovarian, pancreatic, colorectal, stomach, and prostate cancers^12,13^, they remain experimental for many rare cancers, limiting the already scarce treatment options and contributing to lower overall survivals compared to more common tumors^14^. Since the pathognomonic rearrangements in, for example, fusion-driven sarcomas, are generally of diagnostic rather than therapeutic value^15^, and non-rearrangement-driven bone and soft-tissue sarcomas repeatedly demonstrate genomic alterations in various clinically actionable signaling pathways^16–18^ as well as complex biomarkers such as multiple genomic imbalances^18,19^ and deficiencies in homologous DNA repair genes^18,20,21^, a broad molecular analysis, such as whole-exome (WES) or whole-genome (WGS) and transcriptome sequencing (TS), is warranted but is thus far mostly limited to clinical sequencing programs or scientific projects. The Molecularly Aided Stratification for Tumor Eradication Program (DKFZ/NCT/DKTK MASTER) of the German Cancer Research Center (DKFZ), the National Center for Tumor Diseases (NCT), and the German Cancer Consortium (DKTK) is a prospective multicenter clinical sequencing program using WES, WGS, and TS for the identification of therapeutically relevant alterations in patients with rare tumor entities or those under the age of 51 years with advanced tumors and limited or no further treatment options^22–24^. Examination of the first 1310 patients sequenced in the MASTER program and discussed in the multidisciplinary molecular tumor board (MTB) revealed 75.5% rare tumors, corresponding to an incidence of less than six per 100,000 persons per year (https://www.rarecancerseurope.org)^22^. On the other hand, gene panel sequencing is a far more common, heterogeneous sequencing method with a variety of pipelines covering merely a dozen to more than 500 genes and focusing on DNA alterations such as single nucleotide variations (SNVs), small insertions or deletions (indels), and copy number variations (CNVs) without standardized evaluation of homologous recombination deficiency (HRD) or germline assessment, an absence of composite genomic profiles such as mutational signatures, structural alterations, genome duplications^25^ or RNA expression. The TruSight Oncology 500 DNA (TSO500) and TruSight Tumor 170 RNA (TST170) are widely used DNA and RNA sequencing assays covering 523 genes involved in tumorigenesis for SNVs/indels, 59 genes for CNV detection at the DNA level, and 55 genes for the detection of fusions and splice variants at the RNA level^26^.

To date, it is unclear whether WES/WGS, in combination with TS with its obvious additional possibilities has a clinically relevant advantage over panel sequencing in clinical practice owing to a lack of direct comparisons. To address this issue, we conducted TSO500/TST170 panel sequencing of patients who underwent WES/WGS ± TS as participants in the MASTER program using the same tumor tissue and subsequently performed a head-to-head comparison of therapy recommendations (TRs) issued by both WES/WGS ± TS and the panel.

## Results

### Original whole-exome/whole-genome and transcriptome sequencing

**Fig. 1:**
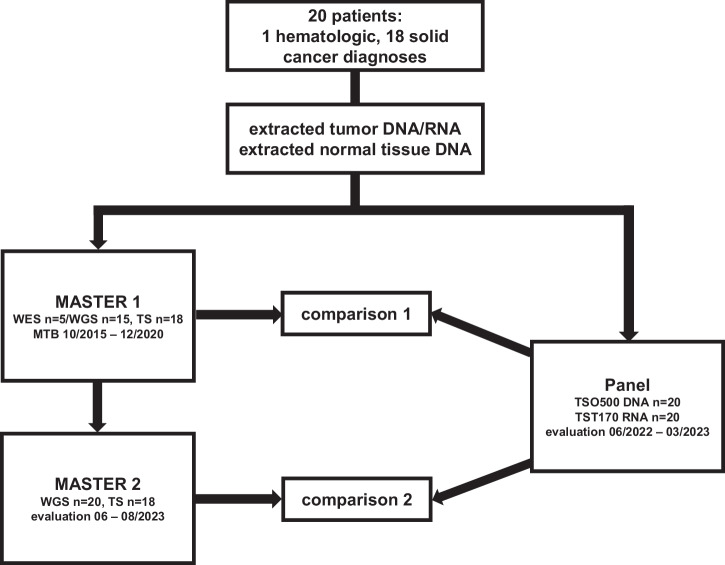
Study flow chart. Extracted tumor DNA and RNA, as well as normal tissue DNA from 20 patients with one hematologic and 18 different advanced solid cancer diagnoses, were originally sequenced as participants in the MASTER program and discussed in the molecular tumor board (MTB) from 10/2015 to 12/2020 (MASTER 1). Aliquots of the same tumor DNA and RNA, as well as normal tissue DNA were then used for panel sequencing (annotation 06–10/2022). To harmonize and update WES/WGS ± TS data, MASTER 1 sequencing data was reanalyzed with the current bioinformatics pipeline of the MASTER program in 06/2023 (MASTER 2). Both panel sequencing and MASTER 2 data were evaluated by physicians trained in precision oncology with access to the patients’ medical history up to the MTB of MASTER 1 (evaluation panel: 06/2022–03/2023, evaluation MASTER 2: 06–08/2023). Therapy recommendations (TRs) and their underlying biomarkers (BMs) derived from the panel were then compared to TRs and underlying BMs from MASTER 1 (comparison 1) and MASTER 2 (comparison 2).

Twenty patients with one hematologic (acute myelomonocytic leukemia [AMML]) and 18 different solid tumor entities (respectively one salivary duct carcinoma, undifferentiated thyroid carcinoma, low-grade ovarian adenocarcinoma, adenocarcinoma of the tongue, adenocarcinoma of unknown primary, adenocarcinoma of the pancreas, acinar cell carcinoma of the pancreas, neuroendocrine carcinoma of the pancreas, mixed neuroendocrine-adenomatous prostate carcinoma, gastrointestinal stromal tumor, leiomyosarcoma, chondrosarcoma, solitary fibrous tumor, desmoplastic small round cell tumor, osteosarcoma, malignant melanoma, glioblastoma, and two cholangiocarcinomas) were enrolled in the DKFZ/NCT/DKTK MASTER program and discussed in a weekly multicenter MTB from 10/2015 to 12/2020 (MASTER 1; Fig. 1; Supplementary Data 1). The median age at the time of MTB was 46 years (range, 25–70 years), and the median number of prior systemic therapies was one (range, 0–8) (Supplementary Data 1). Informed consent was obtained from all patients for tumor and normal tissue sequencing, as well as for further processing and reuse of sequencing and clinical data for research purposes. WES (*n* = 5) or WGS (*n* = 15) and TS (*n* = 18) were performed on extracted tumor DNA and RNA, respectively (Supplementary Data 1). All 20 patients underwent paired germline sequencing of blood DNA for solid tumors (*n* = 19) or saliva for hematologic malignancy (*n* = 1). The clinical relevance of the curated molecular findings was assessed by a team of translational oncologists prior to discussion with the MTB.

### Panel sequencing

Because of the occurrence of distinct alterations at both the DNA and RNA level, aliquots of the same tumor and normal tissue DNA and tumor RNA from the abovementioned 20 patients used for WES/WGS ± TS were resequenced as a validation cohort during the implementation of Illumina’s TSO500 and TST170 assays at the Core Unit for Molecular Tumor Diagnostics at the National Center for Tumor Diseases/University Cancer Center (NCT/UCC) Dresden, Germany, and annotated in 06-10/2022 (Fig. 1). RNA sequencing was successfully performed for all 20 study patients, including the two patients whose TS in MASTER 1 was unsuccessful (patients 3 and 17; Supplementary Data 1). Pipeline extensions, such as enhanced CNV analysis by normalized bin counts and germline assessment by blood/normal tissue pool analysis, are described in the Methods section.

### Reanalysis of whole-exome/whole-genome and transcriptome sequencing

In an effort to homogenize and update WES/WGS ± TS sequencing data, as well as to mitigate temporal differences from the panel analysis, reanalysis of the original sequencing data was conducted using the current MASTER program bioinformatics pipeline in 06/2023 (MASTER 2; Fig. 1). Median time gap between MASTER 1 and MASTER 2 analysis was 4.7 years (range, 2.5–7.7 years; Supplementary Data 1). The main differences between the MASTER 1 and MASTER 2 bioinformatics pipelines are the inclusion of a dedicated germline small variants calling workflow, messenger RNA (mRNA) fusion detection with Arriba version 2^27^ and the change of expression quantification from Reads Per Kilobase Million based on RefSeq gene annotation to Gencode and Fragments Per Kilobase Million calculation. Detailed information on how the state of the art at the time of sequencing (MASTER 1) differs from the uniform MASTER 2 processing is given in Supplementary Data 1. Furthermore, during the nearly eight-year period covered by the data, additional genes of interest and biomarkers were included in line with new clinical studies.

### Comparisons of original/reanalyzed whole-exome/whole-genome and transcriptome data with panel data

TRs and their underlying biomarkers (BMs) issued by physicians from the MASTER sequencing program and discussed in the MTB from 10/2015 to 12/2020 (MASTER 1), as well as TRs from the reanalysis of the original WES/WGS ± TS data (MASTER 2, evaluation 06-08/2023), were juxtaposed with the TRs and underlying BMs from the corresponding panel sequencing data (evaluation 06/2022–03/2023; comparison 1 and comparison 2; Fig. 1). The median time difference was 51.5 months in comparison 1 (range, 18–87 months; Supplementary Data 2) and 6.5 months in comparison 2 (range, 4–14 months; Supplementary Data 2).

### Summary of therapy recommendations and biomarkers

**Fig. 2:**
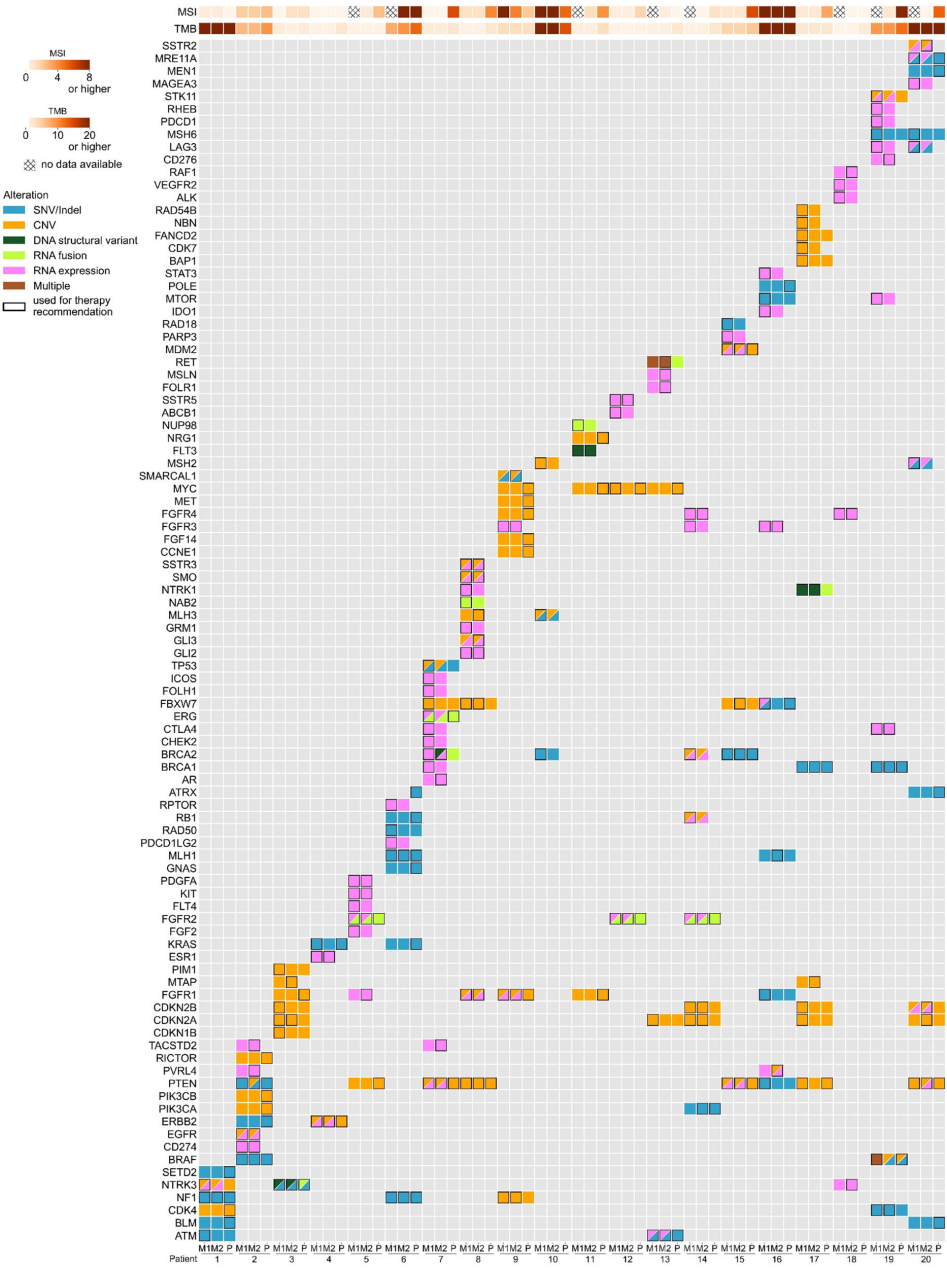
Oncoplot depicting biomarkers of different alteration types used for therapy recommendations (black frame) or their presence (no frame) in both whole-exome/whole-genome ± transcriptome sequencing (WES/WGS ± TS) analyses MASTER 1 (M1) and MASTER 2 (M2) as well as panel sequencing (P) of all 20 study patients. Deletions of NF1 (patient 9), CDKN2A/B (patients 3, 13, 14, 17, 20), and STK11 (patient 19) as well as gains of NTRK3 (patient 1) and PIM1 (patient 3) were all found in a retrospective analysis of panel sequencing data and therefore could not be used as therapy recommendation rationales in P. PVRL4 (patients 2 and 16), TACSTD2 (patients 2 and 7), MTAP (patients 3 and 17), FOLR1 and MSLN (both patient 13) are newer genes of interest in WGS/TS and were all retrospectively analyzed for M1; therefore, they were not available as biomarkers for physicians assessing M1. (MSI microsatellite instability, TMB tumor mutational burden, grid lines: no data available, blue: SNV/Indel [single nucleotide variation/small insertion or deletion], orange: CNV [copy number variation], dark green: DNA structural variant, light green: RNA fusion, pink: RNA expression, dark brown: multiple alteration types of one gene).

In the MASTER 1 analysis, a total of 68 TRs were issued via the original MTB, with a median of 3.5 TRs per patient (range, 1–6). TRs were based on 176 BMs from 14 different categories of alterations: 33 composite biomarkers (high tumor mutational burden [TMB] *n* = 10, increased microsatellite instability [MSI] score *n* = 4, DNA single-base substitution [SBS] mutational signatures^28^ *n* = 10, increased HRD scores^29–31^ *n* = 9), 73 somatic DNA biomarkers (structural variants [SVs] *n* = 3, SNVs/indels *n* = 37, gains/amplifications *n* = 10, deletions *n* = 23), 65 RNA-based biomarkers (fusions *n* = 6, increased mRNA expression *n* = 47, decreased mRNA expression *n* = 12), and five germline biomarkers (germline SNVs *n* = 4, germline deletion *n* = 1; Fig. 2; Supplementary Data 3). The MASTER 2 analysis revealed a total of 61 TRs with a median number of 3.0 TRs per patient (range, 1–6), based on 124 BMs from 14 different categories of alterations: 29 composite biomarkers (high TMB *n* = 7, increased MSI score *n* = 4, SBS mutational signatures *n* = 10, increased HRD scores *n* = 8), 43 somatic DNA biomarkers (SVs *n* = 5, SNVs/indels *n* = 15, gains/amplifications *n* = 7, deletions *n* = 16), 46 RNA-based biomarkers (fusions *n* = 4, increased mRNA expression *n* = 35, decreased mRNA expression *n* = 7), and six germline biomarkers (germline SNVs *n* = 5, germline deletion *n* = 1; Fig. 2; Supplementary Data 3). In the panel data interpretation, physicians recommended a total of 51 TRs with a median number of 2.5 TRs per patient (range, 0–5), based on a total of 75 BMs from seven different categories of alterations: eight composite biomarkers (high TMB *n* = 6, increased MSI score *n* = 2), 57 somatic DNA biomarkers (SNVs/indels *n* = 26, gains/amplifications *n* = 21, deletions *n* = 10), six RNA fusions, and four germline SNVs (Fig. 2; Supplementary Data 3). Molecular evidence levels were assessed according to the classification scheme developed for the MTBs of the NCT and within the DKTK^32^ (Supplementary Data 2).

#### Identical therapy recommendations

**Fig. 3:**
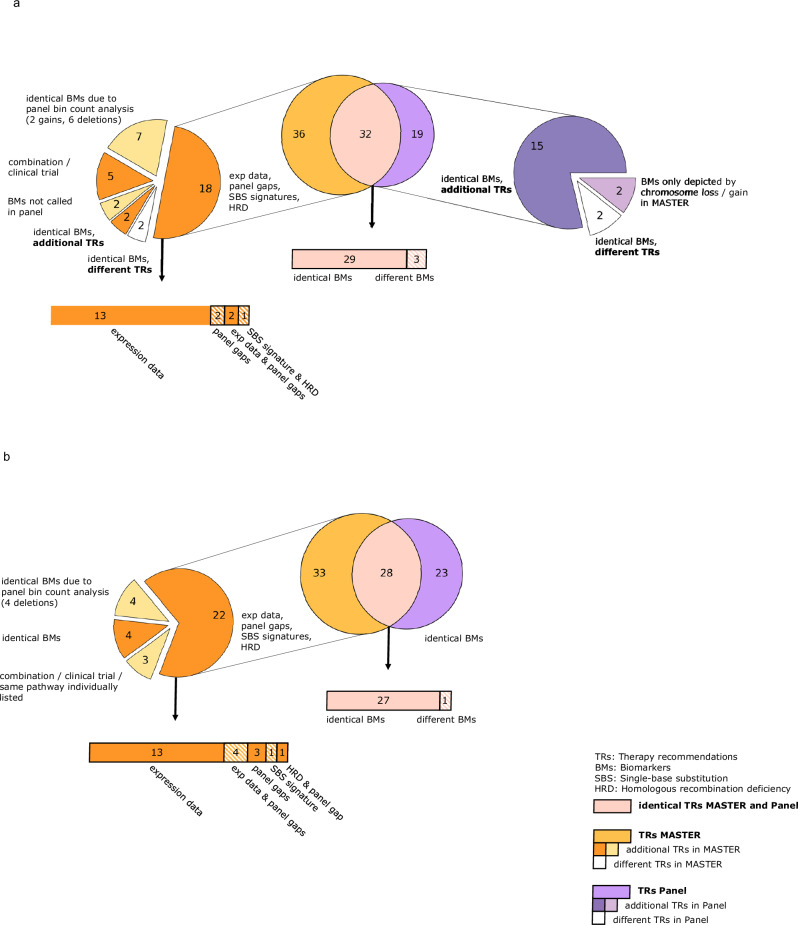
Venn diagrams depicting the sum and categorization of therapy recommendations (TRs) and underlying biomarkers (BMs) in absolute numbers. **a** Comparison 1: original whole-exome/whole-genome ± transcriptome sequencing (WES/WGS ± TS, MASTER 1) versus panel sequencing. **b** Comparison 2: reanalyzed WES/WGS ± TS (MASTER 2) versus panel sequencing. (exp data: RNA expression data, HRD Homologous recombination deficiency, SBS Single-base substitution).

**Fig. 4:**
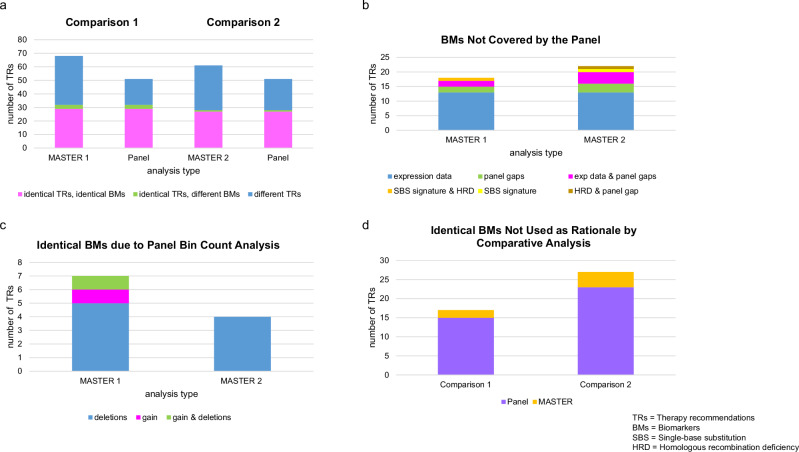
Head-to-head comparison of the most important findings of therapy recommendations (TRs) from the original whole-exome/whole-genome ± transcriptome sequencing (WES/WGS ± TS, MASTER 1), the reanalysis WES/WGS ± TS (MASTER 2), and the panel sequencing. **a** Distribution of identical and different TRs in comparison 1 (MASTER 1 versus panel) and comparison 2 (MASTER 2 versus panel), and their underlying biomarkers (BMs). **b** TRs in MASTER 1 and MASTER 2 based on BMs not covered by the panel. **c** TRs in MASTER 1 and MASTER 2 based on copy number variations retrospectively detected in the panel by bin count analysis. **d** TRs based on identical BMs, which were not used as a TR rationale in the comparative analysis. (exp data: RNA expression data, HRD homologous recombination deficiency, SBS single-base substitution).

In comparison 1, 47.1% (32/68) of TRs from MASTER 1 matched with 62.8% (32/51) of TRs from the panel analysis, with 90.6% (29/32) based on the same BMs and 9.4% (3/32) based on different BM rationales (Figs. 3a and 4a; Supplementary Data 2). In comparison 2, 45.9% (28/61) of the TRs from MASTER 2 aligned with 54.9% (28/51) of the TRs from the panel, with 96.4% (27/28) based on the same BMs and 3.6% (1/28) based on different BMs (Figs. 3b and 4a; Supplementary Data 2).

#### Nonidentical therapy recommendations

In both MASTER 1 and MASTER 2 analyses, 26.5% (18/68) and 36.1% (22/61) of the TRs, respectively, were based on BMs that were not covered by the panel (Figs. 3a, b and 4b; Supplementary Data 2). Of these, 72.2% (13/18) and 59.1% (13/22) were entirely based on RNA expression data, respectively (patients 2, 4, 5, 7, 8, 12, 13, 16, 18–20; Supplementary Data 2 and 4), followed by alterations in genomic regions outside the panel’s target capture which alone or in combination accounted for 22.2% (4/18) and 36.4% (8/22) as well as 5.9% (4/68) and 13.1% (8/61) of all MASTER 1 and MASTER 2 TRs, respectively: In both MASTER analyses, an internal tandem duplication in exon 14 of FMS-like tyrosine kinase 3 (FLT3-ITD) was utilized as the rationale for FLT3 inhibition (patient 11; Supplementary Data 2 and 4). However, the 98 base pairs (bp) long ITD in this patient was not called by the panel since it was covered by only one of the total 24 bins into which FLT3 was divided by the extended CNV calling bin count analysis, and therefore had no effect on the general gene fold change (FC) of 1.1 (Supplementary Fig. 1). Other BMs serving as the basis for therapy, such as amplification of SSTR2 in MASTER 1, amplification of SSTR3, gains of GLI2, GLI3, and PVLR4, homozygous deletions of MTAP, and an SNV in SMARCAL1 in MASTER 2, are generally not covered by the panel (patients 3, 8, 9, 16, 17, and 20; Supplementary Data 2 and 4). In MASTER 1, NAB2:STAT6 and NUP98:NSD1 fusions were used as (co-)BMs for multitargeted tyrosine kinase and Janus kinase inhibition, respectively, whose fusion partners were either not captured by the panel (NAB2, NUP98) or only at the DNA level (STAT6, NSD1) (patients 8 and 11; Supplementary Data 2 and 4). Finally, in both MASTER analyses, the recommendation for PARP inhibition was based on the composite biomarkers mutational signature SBS3 (“BRCAness”^33^) and high HRD scores, which could not be generated by the implemented panel sequencing (patients 3 and 9; Supplementary Data 2 and 4).

In 10.3% (7/68) and 6.6% (4/61) of the TRs of MASTER 1 and MASTER 2, respectively, the underlying BMs were CNVs (two gains and six deletions in MASTER 1 and four deletions in MASTER 2), which could be retrospectively detected in the panel sequencing data by normalized bin count analysis (Fig. 3a, b; Fig. 4c): In both MASTER analyses, germline deletion of exons 40–55 in NF1 served as a rationale for MEK inhibition (patient 9; Supplementary Data 2 and 4), which was not detected by the panel because accurate detection of specific CNVs is affected in the panel’s germline blood pool analysis due to signal dilution. Since TSO500 does not report CNVs for NF1, a retrospective bin count analysis of NF1 in the tumor DNA was conducted and confirmed a somatic NF1 deletion. Furthermore, in both MASTER analyses, four and three TRs of cyclin-dependent kinase 4 and 6 (CDK 4/6) inhibition, respectively, were mainly based on deletion or a codeletion of CDKN2A and CDKN2B (patients 3, 13, 14, 17, and 20; Supplementary Data 2 and 4). The two remaining TRs consisted of NTRK inhibition due to a gain of NTRK3 and mTOR inhibition due to a deletion of STK11 (patients 1 and 19; Supplementary Data 2 and 4).

In both MASTER 1 and MASTER 2 analyses, 7.4% (5/68) and 4.9% (3/61) of the TRs, respectively, were combinations of TRs already listed separately, specific clinical trial recommendations available at the time of evaluation, or the additional listing of a substance targeting the same signaling pathway as another TR already outlined (Fig. 3a, b; patients 2, 8, 10, 14, and 19; Supplementary Data 2 and 4).

Furthermore, 2.9% (2/68) of the TRs obtained from the MASTER 1 analysis in comparison 1 were based on BMs that were not called in the panel (Fig. 3a): In addition to high HRD scores, a recommendation of PARP inhibition was based on a frameshift insertion at the 3’ end of BRCA2 with a tumor variant frequency (TVF) of 0.2, which was detected with a TVF of 0.049 in the panel sequencing and thus was filtered out due to a TVF threshold cutoff of 0.05 (patient 10; Supplementary Data 4–6). A TVF comparison of all called somatic variants in the panel for this patient with the corresponding MASTER 1 data showed consistently lower allele frequencies in the panel despite an average coverage of 1297.1-fold. In contrast, TVF comparisons for two other study patients showed smaller, multilateral deviations (Supplementary Fig. 2 and Supplementary Data 7). A second TR of PARP inhibition in MASTER 1 was built on the “BRCA-like” mutational signature SBS8^34^, low mRNA expression of BRCA2, and a nonfocal loss of chromosome 13, which also contains BRCA2 (patient 14; Supplementary Data 4). Chromosomal bin plot analysis of 13 genes localized on chromosome 13, which are covered by the panel, revealed no significant deletions. The read count of BRCA2 was slightly lower (FC of 0.71), but did not fall below the threshold for deletion calling, which is approximately 0.6 (Supplementary Fig. 3).

In comparison 1, 2.9% (2/68) and 3.9% (2/51) of TRs in MASTER 1 and the panel, respectively, were based on identical BMs, but resulted in different TRs (Fig. 3a): In both cases, a stop-gain mutation in NF1 was used as a rationale for mTOR inhibition in MASTER 1, but served as the basis for MEK inhibition in the respective panel analyses, which were evaluated 43 and 59 months later, respectively, primarily due to new BM-stratified clinical data from other cancer entities (patients 1 and 6; Supplementary Data 2 and 4).

For both comparisons 1 and 2, additional TRs were issued based on identical BMs that were not used as motives for TRs in the comparative sequencing method, accounting for 2.9% (2/68) of MASTER 1 TRs and 29.4% (15/51) of the panel’s TRs in comparison 1, as well as 6.6% (4/61) of MASTER 2 TRs and 45.1% (23/51) of the panel’s TRs in comparison 2 (Fig. 3a, 3b and Fig. 4d; all patients except patients 7, 10, and 18; Supplementary Data 2 and 4).

Finally, 3.9% (2/51) of the additional TRs identified in the panel in comparison 1 were based on BMs depicted only by chromosome loss or gain in MASTER 1 (Fig. 3a): In the first case, the panel-assessing physician recommended mTOR inhibition based on a heterozygous PTEN deletion (FC 0.64); in the second case, a TR of BET inhibition was issued based on a MYC gain (FC 1.42). In both cases, the entire chromosome on which the gene in question was located was affected by the loss or gain, respectively, so that the PTEN deletion and MYC gain were not specifically reported in the MASTER 1 sequencing (patients 5 and 11; Supplementary Data 4).

### Differences in therapy implementations

Ten therapies from nine TRs issued by the MASTER 1 analysis via the MTB could be implemented in a total of eight patients (Table 1). The MASTER 2 analysis recommended eight of the nine MASTER 1 TRs, resulting in nine therapy implementations in the same eight patients (Table 1). One patient with divergent TRs received a recommendation for immune checkpoint inhibition (ICI) in both MASTER 1 and MASTER 2, which led to avelumab as treatment implementation. In addition, MASTER 1, but not MASTER 2 analysis suggested PARP inhibition in this patient, which was implemented by the application of olaparib (patient 20; Table 1; Supplementary Data 3 and 4). The panel sequencing analysis aligned with seven MASTER 1 TRs implemented in six patients, and even provided a second TR of mTOR inhibition in one patient, which was administered in the form of everolimus but was not recommended in neither MASTER 1 nor MASTER 2 analysis (patient 2; Table 1; Supplementary Data 3 and 4). The two remaining patients with therapy implementations in the form of pazopanib received a TR of multitargeted tyrosine kinase inhibition (TKI) from both MASTER 1 and MASTER 2 analyses based primarily on BMs not covered by the panel, that is, RNA expression and a fusion (patient 8; Table 1; Supplementary Data 3 and 4), or RNA expression alone (patient 18; Table 1; Supplementary Data 3 and 4).

**Table 1:** Therapy implementations based on therapy recommendations (TRs) and their underlying biomarkers (BMs) issued in the original whole-exome/whole-genome ± transcriptome sequencing (WES/WGS ± TS; MASTER 1) via the molecular tumor board (MTB). Corresponding TRs and BMs obtained by WES/WGS ± TS reanalysis (MASTER 2) and panel sequencing displayed alongside

	TR MASTER 1	TR MASTER 2	TR panel
**Patient 2**	**BRAF inh** + **MEK inh:** dabrafenib + trametinib	**BRAF inh** + **MEK inh:** dabrafenib + trametinib	**1. BRAF inh** + **MEK inh:** dabrafenib + trametinib
	**BM:** BRAF SNV (V600E)	**BM:** BRAF SNV (V600E)	**BM:** BRAF SNV (V600E)
			**2. mTOR inh:** everolimus
			**BM:** PTEN SNV (C136R), gain in PIK3CA + PIK3CB + RICTOR
**Patient 10**	**ICI:** pembrolizumab	**ICI:** pembrolizumab	**ICI:** pembrolizumab
	**BM:** high TMB, positive MSI sensor, MLH3 SNV (N434fs), foc hom deletion of MSH2	**BM:** high TMB, positive MSI sensor, SBS6 signature	**BM:** high TMB
**Patient 12**	**FGFR inh:** erdafitinib	**FGFR inh:** erdafitinib	**FGFR inh:** erdafitinib
	**BM:** FGFR2:BICC1 fusion	**BM:** FGFR2:BICC1 fusion, high mRNA exp of FGFR2	**BM:** FGFR2:BICC1 fusion
**Patient 14**	**FGFR inh:** infigratinib	**FGFR inh:** infigratinib	**FGFR inh:** infigratinib
	**BM:** FGFR2:WAC fusion, high mRNA exp of FGFR2 + FGFR3 + FGFR4	**BM:** FGFR2:WAC fusion, high mRNA exp of FGFR2 + FGFR4	**BM:** FGFR2:WAC fusion
**Patient 17**	**NTRK inh:** **1**. entrectinib **2**. selitrectinib	**NTRK inh:** **1**. entrectinib **2**. selitrectinib	**NTRK inh:** **1**. entrectinib **2**. selitrectinib
	**BM:** NTRK1-GP2 SV	**BM:** NTRK1-GP2 SV	**BM:** NTRK1-GP2 fusion
**Patient 20**	**1. ICI:** avelumab	**ICI:** avelumab	**1. ICI:** avelumab
	**BM:** high TMB, MSH2 SNV (D580N), low mRNA exp of MSH2, 5x MSH6 SNV (P656L, V11I, S1141F, G670E, T333I)	**BM:** high TMB, SBS15 signature	**BM:** high TMB
	**2. PARP inh:** olaparib		**2. PARP inh:** olaparib
	**BM:** high HRD Scores, MRE11A SNV (R364X), low mRNA exp of MRE11A, foc hom deletion of PTEN		**BM:** MRE11A SNV (R364X), ATRX SNV (E252X), BLM SNV (W1288X), deletion of PTEN
**Patient 8**	**Multi-TKI:** pazopanib	**Multi-TKI:** pazopanib	no
	**BM:** high mRNA exp of FGFR1 + NTRK1, NAB2-STAT6 fusion	**BM:** high mRNA exp of FGFR1, gain in FGFR1	
**Patient 18**	**Multi-TKI:** pazopanib	**Multi-TKI:** pazopanib	no
	**BM:** high mRNA exp of FGFR4 + NTRK3	**BM:** high mRNA exp of FGFR4 + NTRK3 + RAF1	
**TR** = Therapy recommen-dation(s)	**BM** = Biomarker(s)	**SBS** = Single-base substitution	**ICI** = Immune checkpoint inhibition **TKI** = Tyrosine kinase inhibition

Patient 2 with a TR of BRAF and MEK inhibition was treated with dabrafenib and trametinib, patient 20 with a TR of PARP inhibition underwent treatment with olaparib, and patient 17 with a recommendation for NTRK inhibition was treated with both entrectinib and selitrectinib. Patients 10 and 20 with a TR for immune checkpoint inhibition (ICI) were treated with the PD-1 inhibitor pembrolizumab and the PD-L1 inhibitor avelumab, respectively, patients 12 and 14 with a recommendation for FGFR inhibition underwent treatment with erdafitinib and infigratinib, respectively. Patients 8 and 18 both received pazopanib based on a TR of multitargeted tyrosine kinase inhibition (TKI), which was largely or exclusively supported by RNA expression data.

## Discussion

In this study, we performed both WES/WGS ± TS and TSO500/TST170 panel sequencing of patients with rare and/or advanced cancers as participants in the DKFZ/NCT/DKTK MASTER program using aliquots from the same tumor DNA and RNA as well as normal tissue DNA for germline analysis. By directly comparing molecularly informed TRs resulting from both sequencing methods, we evaluated whether WES/WGS ± TS offers a clinically relevant advantage over broad-panel sequencing.

The rate of molecularly informed TRs of the first 1310 patients sequenced in the MASTER program was 86.9%^22^, which was significantly greater than that of panel-based sequencing programs, such as the monocentric MOSCATO 01 trial with a 75-gene panel and a 48.8% TR rate^35^ or the international multicentric WINTHER trial with a 236-gene panel and a 52.1% recommendation rate^36^. However, data from direct comparisons between WGS and comprehensive panel assays are still largely limited. Samson et al. conducted WGS and Ampliseq panel sequencing (52 genes), including Archer Fusionplex analysis (version 1.0 consisting of 33 genes, version 2.0 of 96 genes), and a germline panel (49 genes) of 848 patients with stage IV cancer. Panel sequencing resulted in 36.6% of biomarker-based TRs issued in 250 patients by WGS, which accounted for 343 of 936 TRs in total. A total of 248 therapy options in 190 patients were based on biomarker regions not covered by the panel^37^. To our knowledge, this study is the first direct comparison of the clinical impact of WES/WGS, including TS, with a comprehensive DNA/RNA gene panel covering more than 500 genes.

Notably, the patients in this study were deliberately selected because of their distinct alterations to serve as a validation cohort during the implementation of the Illumina TSO500 pipeline. However, precisely because of the diversity in alteration types, the study cohort may have good validity across various categories of alterations.

Our data demonstrated that 47.1% of the TRs from MASTER 1 in comparison 1 and 45.9% of the TRs from MASTER 2 in comparison 2 matched the TRs from the panel. Furthermore, 26.5% and 36.1% of MASTER 1 and MASTER 2 TRs, respectively, were based on BMs not covered by the panel, with 72.2% and 59.1% of BMs, respectively, consisting of RNA expression data. Notably, two molecularly informed therapies in two patients that were not recommended in the panel analysis were largely or exclusively based on RNA expression data obtained solely by TS. Overall, 22.2% and 36.4% of the TRs not covered by the panel were entirely or partially based on BMs outside the panel’s target capture, which accounted for 5.9% of MASTER 1 TRs and 13.1% of MASTER 2 TRs in total, respectively. The majority consisted of genes that were not covered or fusions consisting of genes that were either not covered or covered only at the DNA level, as well as limitations regarding alterations in covered genes, such as the FLT3-ITD in a patient with AMML, which was covered by a single bin of the panel’s bin count analysis and thus did not affect the general gene FC. Since CNV calling in the panel is determined by the average of the total number of bins, it is clear that focal amplifications and deletions, even if functionally and therapeutically crucial, must comprise far more than a single bin to cause a significant deviation in the FC.

The limitations of the TSO500 panel’s detection of CNVs have become apparent through its restricted reporting of only CNV amplifications of 59 genes, despite over 500 genes being analyzed. This limitation can hinder comprehensive genomic profiling, especially when CNVs outside these 59 genes could provide crucial insights for therapeutic decisions. To address this, we expanded our CNV analysis by visualization of the metadata produced by the Illumina TSO500 pipeline, particularly focusing on normalized bin count analysis. This visualization enables us to extract valuable CNV information beyond the pre-defined gene list. Moreover, to further refine this approach, we introduced a reference line for every analyzed gene based on the mean values derived from approximately 100 samples. This reference helps mitigate the potential for site-specific biases that could otherwise distort the accuracy of CNV calling. Through this expansion, CNV calling using normalized bin count analysis has proven to be a powerful tool, as retrospective analysis identified CNVs that played a key role in therapy selection for 10.3% of patients in MASTER 1 and 6.6% in MASTER 2, underlining the clinical relevance of this assay enhancement.

The remaining TRs in the MASTER analyses outside the panel scope consisted partially or entirely of the DNA mutational signature SBS3 and high HRD scores. Notably, these complex alterations were co-BMs in many other TRs across different categories.

The significant difference between the sequencing methods also becomes evident in the example of a patient with cholangiocarcinoma, whose nonfocal loss of chromosome 13, which also affected BRCA2, was used as a co-rationale for PARP inhibition. As the panel only covers 13 of the total 633 genes localized on chromosome 13^38^, an authentic representation of larger chromosomal changes is not possible. Other inconsistencies, such as the fact that a BRCA2 frameshift insertion in a patient with chondrosarcoma was not called in the panel due to a significantly lower TVF than in MASTER 1, are more likely due to sample preparation issues during the hybridization or enrichment process.

Furthermore, panel sequencing led to a considerable number of TRs (29.4% in comparison 1 and 45.1% in comparison 2) based on BMs that were not used for TRs in the corresponding MASTER analyses. In contrast, only 2.9% and 6.6% of the TRs in MASTER 1 and MASTER 2, respectively, were based on BMs that remained unused for treatment rationales in the panel. This may be due to the greater abundance of therapeutically relevant BMs in WES/WGS ± TS, which even increased in comparison 2 with new genes of interest added to the bioinformatics pipeline for MASTER 2 analysis, which may lead to the prioritization of other, more preferable targets.

In addition to the extended CNV analysis, the complementary germline assessment of 101 genes involved in tumorigenesis is a critical advantage of this study’s panel sequencing, which led to the identification of four out of five pathogenic germline variants, leading to the recommendation of genetic counseling and supporting three TRs. The fifth germline alteration was an NF1 deletion in a patient with neurofibromatosis type 1 and osteosarcoma. Due to germline assessment by blood/normal tissue pool analysis, germline CNVs are concealed by the blending of multiple samples, resulting in this pathognomonic alteration being overlooked^39^. This highlights the limitation of the panel’s germline analysis compared to that of the MASTER program, which comprehensively examines genes associated with tumor predisposition syndromes by single blood/normal tissue analysis^24^.

In summary, this study revealed that WES/WGS and TS have advantages over large panel-based sequencing, mainly because of the presence of RNA expression, the unlimited number of covered genes, and composite biomarkers, such as DNA mutational signatures and HRD scores, which can expand the limited treatment options available for patients with rare and advanced tumors. The advantages of panel sequencing, such as simpler logistics due to the possibility of using archived, formalin-fixed instead of fresh tumor tissue, a generally shorter turnaround time from the start of DNA/RNA extraction to molecular results compared to WGS/TS, as well as generally lower costs, are of a more practical nature but are nevertheless important for everyday clinical practice. Furthermore, in our experience, the drop-out rates for TSO500 DNA/TST170 RNA panel sequencing are comparable to those for WGS and RNA sequencing, provided that the pathological tumor cell content of the fresh frozen tissue is at least 20%, a condition met in most cases. All in all, to conclusively assess the actual socioeconomic and clinical impact of choosing WES/WGS + TS over broad-panel sequencing, further randomized controlled trials with larger patient cohorts are needed.

In conclusion, WGS, with its additional layers of complex biomarkers such as aneuploidy, viral integration, the possibility of discovering potential novel chromosomal rearrangements^40^, the addition of RNA sequencing, the integration of DNA methylation profiling for entity prediction^41^, (phospho-)proteomics^42^, pharmacogenomics, and preclinical drug testing in patient-derived organoids, spheroids, and cell cultures into the MTB discussion, enables a multidimensional tumor characterization beyond the capacities of a predefined panel. This approach offers the possibility to investigate new genes of interest, genomic patterns of tumorigenesis, and drug resistance and may, therefore, significantly improve the development of effective targeted therapies in the future.

## Methods

### Ethical approval

This study was conducted in accordance with the principles of the Declaration of Helsinki. All 20 participating patients provided written informed consent for the banking of tumor and control tissues, molecular analysis, and collection of clinical data under a protocol approved by the Ethics Committee of the Medical Faculty of Carl Gustav Carus University Dresden and Heidelberg University. By agreeing to participate in the DKFZ/NCT/DKTK MASTER program, the patients consented to the publication of their own pseudonymized data in the event of future publications.

### Whole-exome/whole-genome and transcriptome sequencing

Bioinformatic analyses of the sequencing data were performed based on the corresponding bioinformatics pipelines at the time of patient enrollment as previously described by Horak et al.^22^. The WES/WGS ± TS datasets were annotated and curated by the program’s bioinformaticians. Comprehensive somatic and germline sequencing data, categorized according to different alteration types and underlying bioinformatics pipelines, were provided in an Excel file. Additionally, an overview of potentially relevant findings, including suspected driver alterations and known targetable lesions, was provided to facilitate structured interdisciplinary discussions within the MTB. As of 09/2016, composite markers such as SBS mutational signatures^28^, and as of 11/2018, composite markers related to HRD, such as HRD scores^29–31^, have been identified alongside the evaluation of somatic and germline alterations in DNA damage response genes.

### Panel sequencing

For targeted sequencing, 40–120 ng of each total DNA and RNA sample was used. Library preparation was performed using the TSO500 Kit (Illumina, San Diego, CA, USA) according to the manufacturer’s protocol. Barcoded libraries were sequenced (2 × 150 bp paired-end) on an Illumina NextSeq 500 platform. Raw sequencing data were analyzed using the Illumina TSO500 Local App version 2.2.0.2 in a Docker container, with GRCh37 serving as the reference genome, followed by an in-house developed extended downstream analysis. The resulting small variants were subsequently annotated using gnomAD (version 2.1.1)^43^, ClinVar (version 10_07_2023)^44^, and COSMIC (version 09_06:2022)^45^. The filtration and effect of the variants were predicted using SnpEff^46^ and SnpSift [4.3t]^47^. In addition to the CNV results provided by Illumina, an enhanced CNV analysis was performed by analyzing the raw data from Illumina’s CNV Caller, a method used to extend CNV calling in the panel as TSO500 reports CNVs for only 59 genes. Therefore, the normalized bin counts for each gene, dividing the gene into an individual number of bins comprising 50–250 bp as provided by the TSO500 pipeline, were plotted against an internal in-house reference line. To identify germline variants, a blood pool or a pool of normal, nontumor tissue, was utilized using the patient’s own blood or normal tissue samples. The advantage of blood/normal tissue pool analysis is the ability to distinguish between germline variants that are detected in both tumor and blood/normal tissue DNA and somatic variants that are solely detected in the tumor DNA. On the other hand, sample pooling leads to a loss of individual information and dilution of signals, which results in limitations in the detection of rare germline variants, particularly those with low allelic frequencies and germline variants affected by loss of heterozygosity. Furthermore, detecting CNVs in a pooled sample is challenging or unfeasible because pooling can obscure copy number variations in individual samples. In addition to the TST170 fusion pipeline, mRNA fusions were analyzed using Arriba (version 2.4.0)^27^.

### Evaluation of panel and reanalyzed whole-exome/whole-genome and transcriptome sequencing

Translational oncologists assessing the panel sequencing and the WES/WGS ± TS reanalysis (MASTER 2) were granted access to the patients’ medical history up to the point of the original MASTER 1 MTB, but had no insight into the original WES/WGS ± TS data or corresponding TRs. The evaluation of TRs was based on the current state of research at the respective time of assessment (evaluation panel: 06/2022-03/2023, evaluation MASTER 2: 06-08/2023; Fig. 1). The resulting TRs were discussed with a senior physician in the department. Corresponding patient sequencing data from the panel and the MASTER 2 analysis were not evaluated by the same physician.

## Supplementary information

Supplementary Information

Supplementary Data 1

Supplementary Data 2

Supplementary Data 3

Supplementary Data 4

Supplementary Data 5

Supplementary Data 6

Supplementary Data 7

## Data Availability

The sequencing data generated are deposited and available through the European Genome-Phenome Archive (EGA) under the study identifier: EGAS50000000431. Whole-genome and transcriptome versus panel sequencing in precision oncology: a translational-clinical comparison.
