# Supplementary material for: Translational and clinical comparison of whole genome and transcriptome to panel sequencing in precision oncology
Source: NPJ Precis Oncol. 2025 Jan 10;9:9. doi: 10.1038/s41698-024-00788-3 (PMC11724059; doi:10.1038/s41698-024-00788-3)

Supplementary Information

In order of appearance in the manuscript:

Supplementary Data 1 (excel file, uploaded separately). Patient characteristics and overview of sequencing methods applied to each patient. Changes of bioinformatics pipeline between the two different WES/WGS ± TS analyses (MASTER 1 and MASTER 2) for each patient (sheet 1) and in general (sheet 2).

Supplementary Data 2 (excel file, uploaded separately). Analysis and juxtaposition of therapy recommendations (TRs) issued in the original whole-exome/whole-genome ± transcriptome sequencing (WES/WGS ± TS, MASTER 1) and the panel (comparison 1 [sheet 1]), as well as in the WES/WGS ± TS reanalysis (MASTER 2) and the panel (comparison 2 [sheet 2]), broken down into subtypes according to biomarker (BM) properties and availability. Detailed presentation of the BMs of the TR categories “TRs MASTER based on expression (exp) data, panel gaps, SBS mutational signatures, HRD” and “identical BMs due to panel bin count analysis leading to additional (add.) TRs in MASTER”. (SBS: Single-base substitution, HRD: Homologous recombination deficiency). Sheet 3: Molecular evidence levels of all MASTER 1, MASTER 2 and panel TRs. Sheet 4: Patient outcomes of patients with molecularly informed therapy implementations listed in Table 1.

Supplementary Data 3 (excel file, uploaded separately). Summary of therapy recommendations (TRs) and therapy recommendations leading to therapy implementations (TR Imps) in the original whole-exome/whole-genome ± transcriptome sequencing (WES/WGS ± TS, M1/MASTER 1 [sheet 1]), WES/WGS ± TS reanalysis (M2/MASTER 2 [sheet 2]), and panel (P/Panel [sheet 3]) broken down by biomarker type.

Supplementary Data 4 (excel file, uploaded separately). Sheets 1-20: Detailed presentation of treatment recommendations (TRs) and corresponding biomarkers (BMs) of the original whole-exome/whole-genome ± transcriptome sequencing (WES/WGS ± TS, M1), the WES/WGS ± TS reanalysis (M2), and the panel of each study patient displayed adjacent to each other. Summary of the juxtaposition of TRs issued in M1 and the panel (comparison 1) as well as in M2 and the panel (comparison 2). Information on the presence of a germline BM in the WES/WGS ± TS leading to the recommendation of genetic counseling and whether the BM was detected in the panel.

Supplementary Figure 1 (attached in PDF file). Panel bin plot analysis of the FLT3 gene of patient 11. The tandem duplication in FLT3 (FLT3-ITD) detected by the original and reanalyzed whole-exome sequencing (MASTER 1 and MASTER 2) affects only one bin (highlighted in green), which does not result in a positive deflection of the general fold change (FC); therefore, no FLT3 alteration was reported.

Supplementary Data 5 (excel file, uploaded separately). Insertions and deletions (indels) detected in the original whole-genome + transcriptome sequencing (WGS + TS, MASTER 1) of patient 10. BRCA2 frameshift insertion relevant for TR in MASTER 1 is highlighted in yellow (row 98), the tumor variant frequency was 0.2 (column AC).

Supplementary Data 6 (excel file, uploaded separately). Small variants, including small nucleotide variants (SNVs) and insertions/deletions (indels) in the panel sequencing data of patient 10. The BRCA2 frameshift insertion relevant for TR in the original whole-genome + transcriptome sequencing (WGS + TS, MASTER 1: NM_000059.3:c.7177dup) was also detected by the panel (highlighted in yellow, row 980), yet with a significantly lower tumor variant frequency of 0.0485 (column F).

Supplementary Figure 2 (attached in PDF file). Box plots demonstrating the differences in allelic frequencies (tumor variant frequency [TVF]) of common somatic variants called in the panel (TVF > 0.05) and in the whole-genome sequencing of MASTER 1 of patient 10 (green). Equivalent TVF comparisons of patients 1 (red) and 12 (blue) functioning as controls. Whereas for patient 10, TVFs are unilaterally lower in the panel with differences ranging from 0.077 to 0.398, TVF comparisons for patients 1 and 12 showed smaller, multilateral deviations ranging from -0.147 to 0.178 and -0.064 to 0.094, respectively.

Supplementary Data 7 (excel file, uploaded separately). Comparison of the tumor variant frequencies (TVFs) of common somatic variants identified in the panel (TVF > 0.05) and the original whole-genome + transcriptome sequencing (WGS + TS, MASTER 1) of patient 10 (sheet 1). Sheets 2-3: Equivalent TVF comparisons of patients 1 and 12 as controls.

Supplementary Figure 3 (attached in PDF file). Chromosomal bin plot analysis of 13 genes localized on chromosome 13 covered by the panel of patient 14. BRCA2 shows a slightly decreased fold change (FC) of 0.71, which does not lead to deletion calling (FC cutoff ≤ 0.6).

Supplementary Figure 1

TSO500, patient 11: FLT3 bin plot analysis

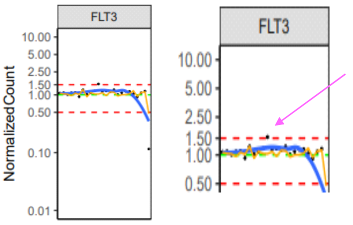

chromosome	start	end	GC amount	gene	bin name	raw reads	normalized bin count (FC)	bin length (bp)
chr13	28578187	28578313	0,511811023622047	FLT3	chr13.50	1097	1,072537756611610	126
chr13	28588587	28588696	0,445454545454545	FLT3	chr13.51	920	1,088267446173980	109
chr13	28589292	28589395	0,375000000000000	FLT3	chr13.52	799	1,010743516424330	103
chr13	28589725	28589840	0,465517241379310	FLT3	chr13.53	1010	1,084748254802500	115
chr13	28592602	28592728	0,496062992125984	FLT3	chr13.54	1271	1,099046166862750	126
chr13	28597485	28597616	0,356060606060606	FLT3	chr13.55	763	0,929682539220965	131
chr13	28598996	28599082	0,471264367816092	FLT3	chr13.56	530	1,224957299290440	86
chr13	28601223	28601380	0,348101265822785	FLT3	chr13.57	862	1,014611958998910	157
chr13	28602313	28602427	0,513043478260870	FLT3	chr13.58	967	1,086521023506860	114
chr13	28608022	28608130	0,440366972477064	FLT3	chr13.59	999	1,168450727671390	108
chr13	28608217	28608353	0,386861313868613	FLT3	chr13.60	1421	1,543872197324050	136
chr13	28608436	28608546	0,405405405405405	FLT3	chr13.61	900	1,143782427988240	110
chr13	28609630	28609812	0,453551912568306	FLT3	chr13.62	1242	1,168611851741860	182
chr13	28610070	28610182	0,513274336283186	FLT3	chr13.63	974	1,129234032574220	112
chr13	28611320	28611427	0,361111111111111	FLT3	chr13.64	685	1,227936921815580	107
chr13	28622410	28622582	0,364161849710983	FLT3	chr13.65	756	1,009445415813360	172
chr13	28623519	28623676	0,417721518987342	FLT3	chr13.66	1123	1,086174588631980	157
chr13	28623770	28623913	0,430555555555556	FLT3	chr13.67	1032	1,151033988231350	143
chr13	28624230	28624361	0,439393939393939	FLT3	chr13.68	1075	1,148252788334480	131
chr13	28626680	28626813	0,477611940298507	FLT3	chr13.69	970	1,041203429788820	133
chr13	28631482	28631601	0,350000000000000	FLT3	chr13.70	708	0,917883421311713	119
chr13	28636002	28636208	0,531400966183575	FLT3	chr13.71	1880	1,085013324668480	206
chr13	28644626	28644751	0,349206349206349	FLT3	chr13.72	563	1,160135014618250	125
chr13	28674603	28674649	0,787234042553191	FLT3	chr13.73	8	0,116065330795185	46
							average FC: 1,1	

bin affected by internal tandem duplication

The FLT3-ITD* found in MASTER is only 98 base pairs in size. It is covered by a single bin (28608217-28608353) of the CNV caller.
The FC of this bin is 1.5, which is slightly above the upper border for duplications (arrow pointing at respective dot in CNV plot).
Thus, this duplication escaped detection as it is too small.
By normalized bin plot analysis, we would have **not** reported a **FLT3 internal tandem duplication** (gene FC=1.1).

FLT3-ITD: internal tandem duplication of the FMS-like tyrosine kinase 3

FC: fold change

Supplementary Figure 2

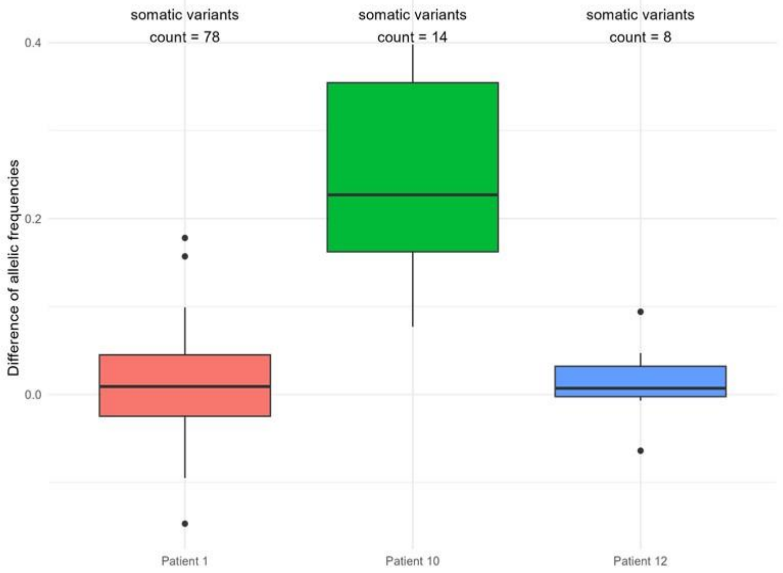

Supplementary Figure 3

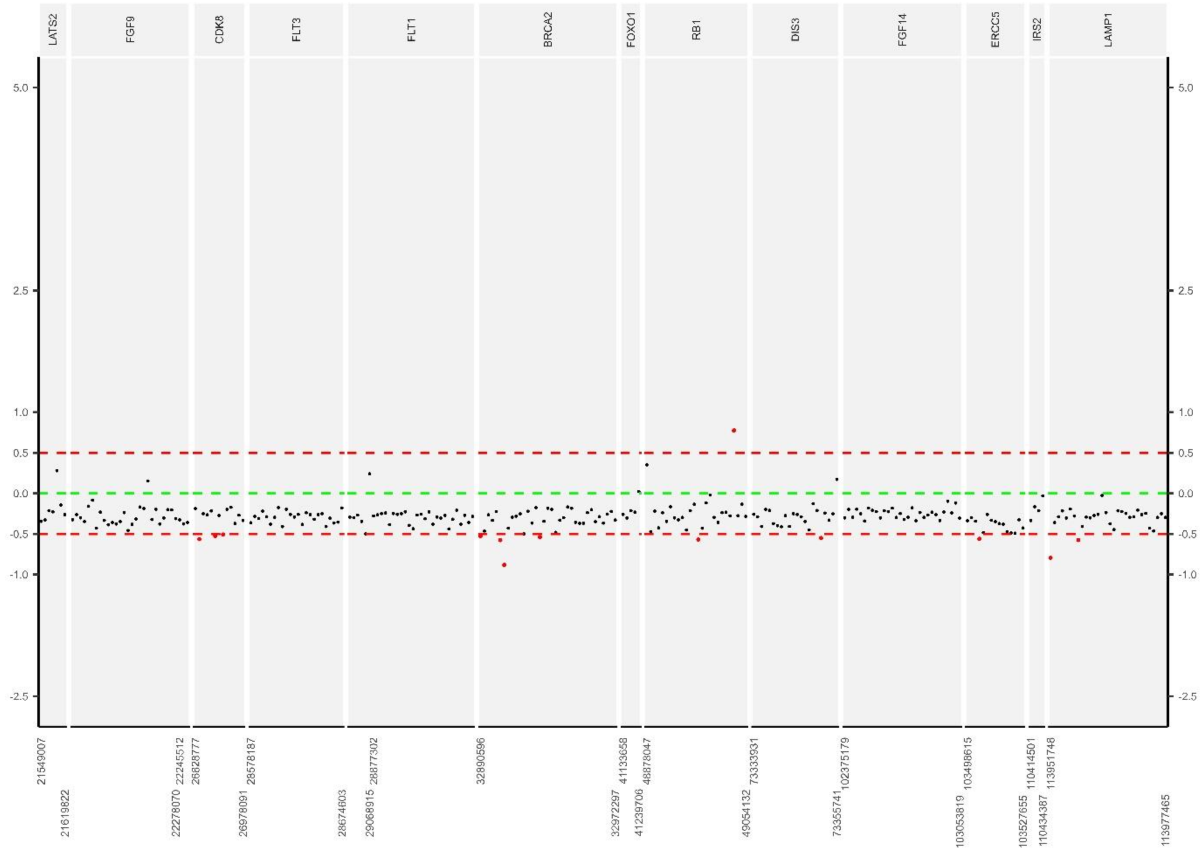

Supplement: Supplementary file 1 — Supplementary Information [file 41698_2024_788_MOESM1_ESM.pdf]
